# Extending the Targets for Coronavirus Antivirals Beyond That of Approved Drugs: Insights From Preclinical Research

**DOI:** 10.1111/1751-7915.70376

**Published:** 2026-05-22

**Authors:** Harald Brüssow

**Affiliations:** ^1^ Department of Biosystems, Laboratory of Gene Technology KU Leuven Leuven Belgium

## Abstract

Antiviral drugs have been approved for the treatment of COVID‐19. However, they present pharmacological limitations, a mixed efficacy profile and target just two coronavirus proteins. To extend the range of druggable coronavirus proteins, researchers explored small molecule N‐glycan binders as inhibitors of SARS‐CoV‐2 spike protein interaction with the cell receptor. Other groups investigated lipopeptides as inhibitors of cell fusion by viral spikes. High throughput screening of chemical libraries yielded viral maturation inhibitors that targeted the viral M protein. Massive screening led to inhibitors of the non‐structural coronavirus protein NSP14, a methyltransferase involved in viral mRNA cap synthesis. Machine learning–driven scans of chemical space revealed inhibitors of non‐structural coronavirus protein NSP3, a papain‐like protease subverting innate immune response to viral infection. A chimera of a nucleotide analogue coupled to an RNase L attractor bound the RNA‐dependent RNA polymerase NSP12 and mediated degradation of the viral RNA. Several of these compounds showed comparable or even superior antiviral efficacy as approved COVID‐19 drugs in preclinical animal tests. Parallel efforts were made to develop chemical compounds targeting host proteins needed for viral multiplication. Peptidomimetic tetrapeptides acted as inhibitors of the host protease TMPRSS2 involved in cell fusion by the viral spike protein. A repurposed TMPRSS2 inhibitor was tested in COVID‐19 patients without demonstrating efficacy. A genetic screen demonstrated an enzyme involved in sphingomyelin synthesis and its inhibitor which impaired SARS‐CoV‐2 replication. A viral‐cell protein interactome study showed 332 cellular proteins interacting with 26 coronaviral proteins. A chemoinformatic search found inhibitors for the interaction of NSP9 with host elongation factor eIF4A and for NSP13 with elongation factor eEF1A. Plitidepsin, a clinically used eEF1A inhibitor, was tested in human clinical trials with COVID‐19 patients demonstrating in vivo antiviral activity and a trend for clinical amelioration in an underpowered phase 3 clinical trial.

## Graphical abstract

Chemical structure of RIBOTAC inhibitor with metabolic handle binding as nucleotide analogue to SARS‐CoV‐2 RNA dependent RNA polymerase, its linker and the RNase L recruiter which binds RNase L monomers and mediates their dimerization that actives nuclease activity degrading the viral RNA. Figure credit: Min et al., 2024 under a Creative Commons Attribution License 4.0.

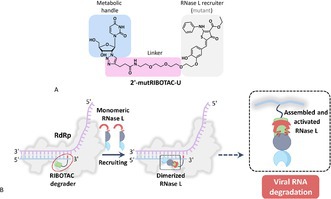

The COVID‐19 pandemic has revealed a critical lack of antiviral drugs in general and in particular against this newly emerging viral pathogen. Early in the COVID‐19 pandemic and in parallel with vaccine development efforts, SARS‐CoV‐2 antiviral drug research became a focus of scientific activities. Several antiviral drugs for the treatment of COVID‐19 were indeed soon approved by health authorities. This should however not be a reason for complacency. The basic research work leading to these drugs was conducted many years before the onset of the COVID‐19 pandemic and it initially targeted other viral pathogens. Without this preparative work, antiviral drugs would hardly be at hand during the pandemic, stressing the importance of ongoing antiviral drug research as a source for antivirals should we confront a new viral pandemic. As zoonotic coronavirus infections, particularly from bats, remain a concern, such antiviral drugs should not only inhibit SARS‐CoV‐2 but also a wider range of coronaviruses. There are other reasons why antiviral research should remain a priority even if the COVID‐19 pandemic is changing into an endemic where SARS‐CoV‐2 might join other coronaviruses causing common cold syndromes in the future. First, SARS‐CoV‐2 might evolve new variants with high pathogenicity escaping protection with current vaccines. Second, post‐acute sequelae of SARS‐Cov‐2 (PASC), also called long‐Covid, remain a major public health challenge for which no drugs have been developed (although it remains unclear whether antivirals could here play a role). Third, the approved COVID‐19 antivirals have disadvantages. Either they need intravenous application (remdesivir) or they need a combination with a drug booster (nirmatrelvir with ritonavir), leading to drug–drug interactions, or they raised potential concerns about teratogenic effects in pregnant women and accelerating viral evolution (molnupiravir). In addition, clinical trials indicate variable clinical efficacy of these coronavirus antivirals. More efficient antiviral drugs would therefore be needed. Fourth, only two coronaviral non‐structural proteins (NSP) are targeted by approved COVID‐19 antivirals: the RNA‐dependent RNA polymerase (RdRp; NSP12) by remdesivir and molnupiravir and the main protease (Mpro, also referred to as chymotrypsin‐like protease 3CLpro; NSP5) by nirmatrelvir (Figure 1). Other coronavirus functions should be explored as targets for new antivirals since therapies using a single antiviral drug can lead to resistance developments. Combination therapies with antivirals targeting several coronavirus proteins could reduce the risk of resistance development. Fifth, antivirals targeting host proteins essential for viral infections are seriously underexplored, not only for coronaviruses but as an antiviral approach in general. The current article reviews preclinical research addressing the last two points: extending the antiviral targets to further coronavirus proteins and functions and exploring antivirals directed against host cell proteins. The article also raises the question of whether broadly cross‐reacting antivirals active against different viral families are feasible.

**FIGURE 1:**
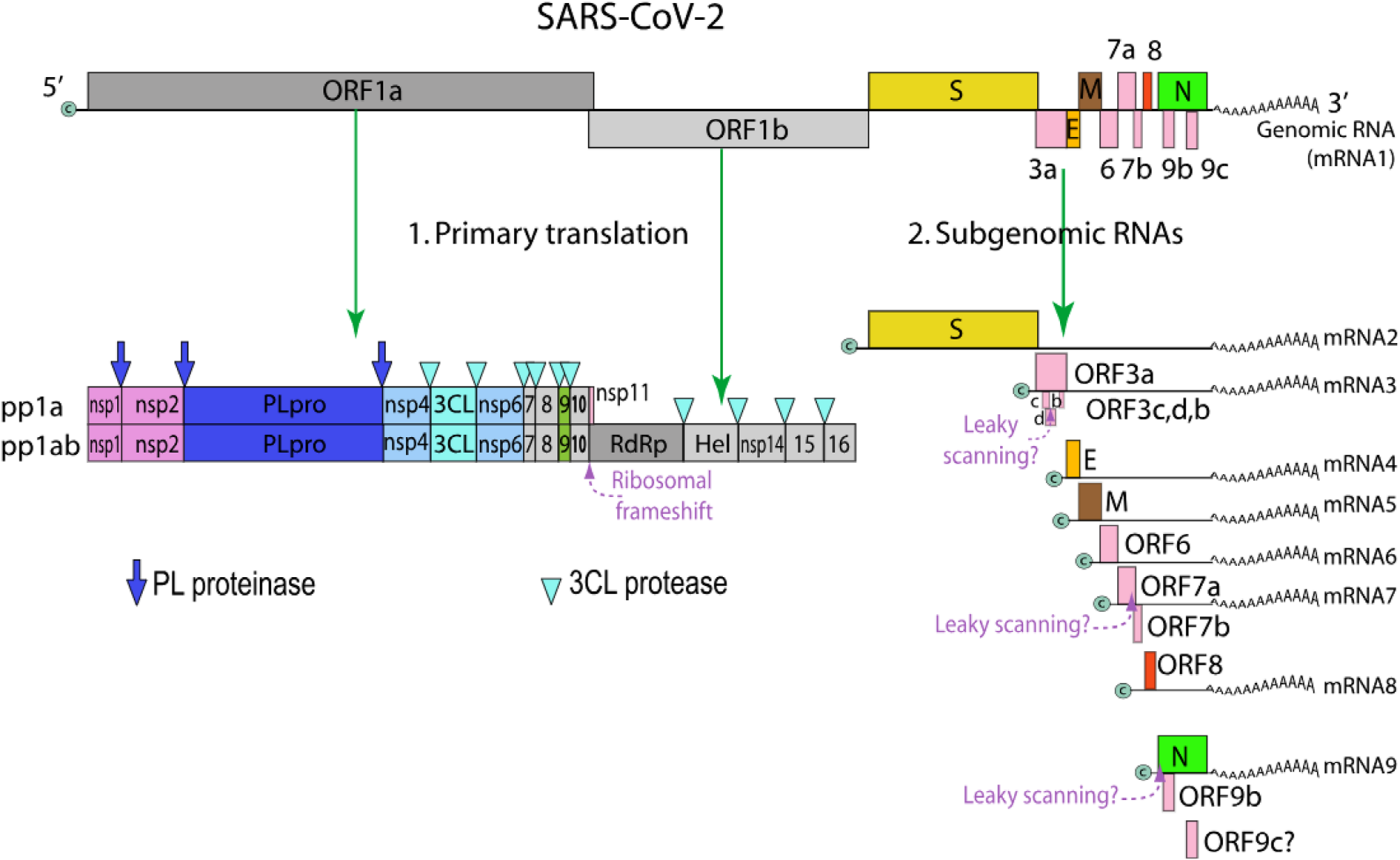
SARS‐CoV‐2 genome map with primary polyprotein translation products and cleavage into non‐structural proteins NSP1 to NSP16 by PL protease activity (a domain in the multidomain protein NSP3) and 3 CL protease (NSP5) as indicated by blue arrow (PLpro) and light blue wedge (3CLpro). The spike protein S and the M protein mentoned in the text are translated from subgenomic RNAs. Figure credit: ViralZone from Swiss Institute of Bioinformatics under a Creative Commons Attribution 4.0 International Licence.

## 1 Targeting Viral Functions With Antivirals

### 1.1 N Glycan Binders of Viral Spikes as Viral Attachment Inhibitors

**FIGURE 2:**
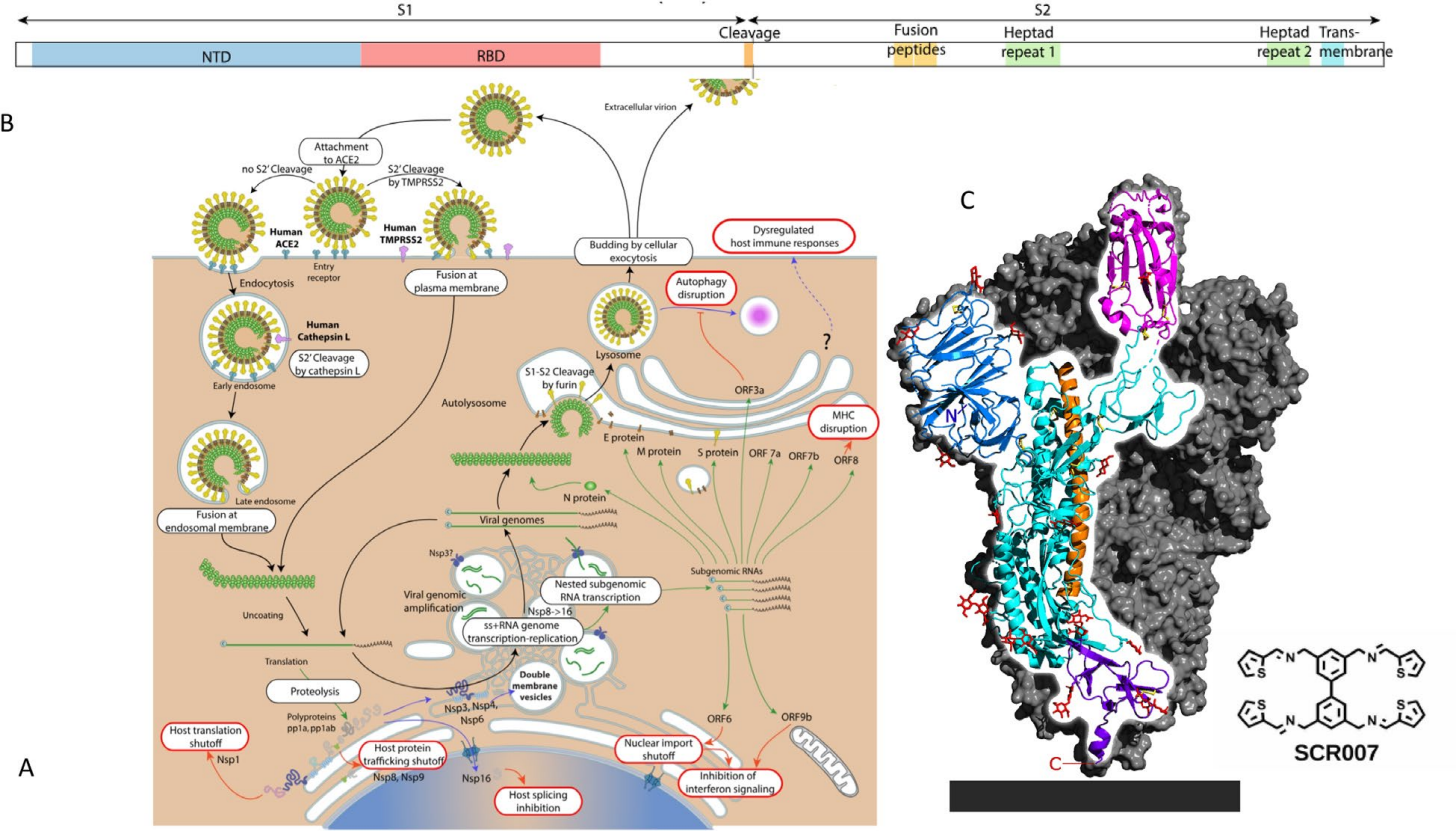
SARS‐CoV‐2 cell recognition, fusion and replication cycle. (A) Overview of SARS‐CoV‐2 cell infection, replication and maturation. (B) Structure of the spike protein with N‐terminal domain (NTD), receptor binding domain (RBD), proteolytic cleavage site into S1 and S2 subunits and adjacent to the cleavage site the fusion peptide. (C) Structure of the spike protein trimer, one monomer is coloured: Blue (NTD), magenta (RBD), red (glycosylations), grey block at bottom (viral membrane. Insert: Structural formula of the N‐glycan binder described by Ezzatpour et al. (2025)). Figure credit: ViralZone from Swiss Institute of Bioinformatics under a Creative Commons Attribution 4.0 International License (A, B), Wikipedia 5‐HT2AR, public domain (C).

US researchers argued that an antiviral with broad‐spectrum activity against a range of enveloped RNA viruses with pandemic potential such as coronaviruses, paramyxoviruses and filoviruses would be a crucial drug in case of a future pandemic before specific vaccines against the new disease could be developed. The sequence diversity found in viral proteins within and between viral groups and their rapid evolution in face of the human immune system makes the development of antivirals directed against viral proteins a challenging task. These researchers hypothesized that the N‐linked glycans of the viral spike proteins are a suitable target due to their conserved Manα1–3(Manα1–6)Manβ1–4GlcNAcβ1–4GlcNAcβ(GlcNAc_2_Man_3_) pentasaccharide core across viruses (Figure 2). They developed a chemical library of 57 synthetic carbohydrate receptors (SCR): around a conserved biaryl central structure four different side chains with different heterocycles were synthesized (Figure 2C, insert). Several SCRs displayed inhibitory activity against viral pseudotypes representing five of the six potential pandemic viral strains belonging to three different viral families. In neutralization tests with authentic viruses, one SCR displayed inhibitory activity in the low micromolar (μM) concentration range. Upon intranasal and intraperitoneal SCR application, no adverse effects were observed in mice which is important since N‐linked glycans are also found on cellular glycoproteins. When SCR007 was given together with SARS‐CoV‐2 to mice, 90% of the animals survived the infection while all mice receiving an inactive SCR died. At time of death in controls, SCR007‐treated mice showed practically no viral RNA by RT‐PCR in lung and brain tissue and no pathology. By time of addition analysis, the researchers determined that SCR007 inhibited viral binding but had no effect on the subsequent fusion step. Nuclear magnetic resonance (NMR) spectroscopy confirmed the specific binding of SCR007 to the RBD of the SARS‐CoV‐2 spike protein. SCR007 exhibited binding affinity towards fucosylated complex‐type tri‐and tetra‐antennary N‐glycans, with selectivity for those on viral glycoproteins (Ezzatpour et al. 2025).

Italian scientists had developed alternative SCRs based on a benzene ring substituted by six aminopyrrolic side groups that bound the highly mannosylated envelope glycoprotein gp120 of the human immunodeficiency virus (HIV). One such compound also showed dose‐dependent inhibition of SARS‐CoV‐2 pseudoviruses representing different variants of concern, as well as authentic SARS‐CoV‐2 in cell culture. They demonstrated that the compound bound the RBD of the viral spike protein, that it showed no interaction with the cellular ACE2 glycoprotein host receptor and competed with RBD binding to the ACE2 receptor (Francesconi et al. 2022).

Broad spectrum antiviral activities had previously also been documented for lectins and peptides, but their high molecular weight and protein nature have hampered the development of drugs. The antiviral protein griffithsin isolated from a red alga displays three nearly identical carbohydrate binding domains that react with high mannose oligosaccharides on HIV envelope glycoprotein gp120. In cell culture it inhibits infection with several human and animal coronaviruses. It binds SARS‐CoV‐1 glycoprotein at multiple sites with high affinity. In a mouse infection model, griffithsin given concomitantly with SARS‐CoV‐1 achieved 100% survival while control mice showed 75% mortality. Griffithsin reduced viral load only moderately but attenuated the cytokine response (O'Keefe et al. 2010). Also, the lectin FRIL from an edible bean selectively binds to complex‐type N‐glycans and neutralizes viruses in cell culture that possess complex‐type N‐glycans on their envelopes including SARS‐CoV‐2 (Liu et al. 2020).

### 1.2 Lipopeptides as Viral Fusion Entry Inhibitors

The spike protein S of SARS‐CoV‐2 shows a prefusion conformation in the outer membrane of the virion. Upon attachment to the host cell receptor and proteolytic processing into S1 and S2 viral spike subunits, the fusion peptide at the viral S2 cleavage site becomes inserted into the cellular membrane (Figure 2B). Via association of two heptad repeats of the S2 subunit, the protein rearranges into a compact six‐helix bundle that drives the fusion of host and viral membranes (Figure 2A). US researchers demonstrated that a lipid‐conjugated peptide corresponding to the C‐terminal heptad repeat blocks the infection of cell cultures with SARS‐CoV‐2 (Outlaw et al. 2020). Subsequently, they optimized the inhibitor. Optimal inhibition was achieved by a dimeric heptad peptide linked by four polyethylene glycol (PEG_4_) units to which a cholesterol was conjugated. This molecule showed inhibitory activity at low nM concentration in Vero cells expressing the cellular TMPRSS2 protease. Here a short comment on this cell line: The Vero cell lineage was isolated from kidney epithelial cells of an African green monkey (*Cercopithecus aethiops*, now renamed Chlorocebus sabaeus) by Japanese researchers in 1962 at Chiba University. The immortalized cell lineage is continuous and aneuploid. The whole genome was sequenced (Osada et al. 2014) revealing a homozygous 9 Mb deletion in chromosome 12 covering the type I interferon gene cluster and cyclin‐dependent kinase inhibitors. The sequence showed that it was derived from a female animal. The genetic deficiency in the production of interferon I renders the cell line susceptible to infection with a broad range of viruses making Vero cells a standard cell line for virological research. Vero cells were approved by WHO and FDA for vaccine production and thus also became the most widely used continuous cell line for the production of vaccines over the last two decades. Vero E6 cells are also highly susceptible to SARS‐CoV‐2 infection due to high expression of the ACE2 receptor. However, it lacks the serine protease TMPRSS2 involved in cell entry (Figure 2A). Therefore transformed Vero E6/TMPRSS2 cells are frequently preferred for in vitro tests since this cell line enhances the cleavage of the viral spike protein and thus more closely mimics human airway cell infection.

Upon intranasal instillation of the lipopeptide inhibitor to mice, high antiviral concentrations with long persistence were observed in the lungs. The researchers tested the in vivo prophylactic efficacy of this lipopeptide in naïve ferrets that were cohoused with SARS‐CoV‐2 infected ferrets. Exposed naïve ferrets receiving SARS‐CoV‐2 specific lipopeptides showed no viral RNA in throat or nose, no infectious virus was isolated, and no seroconversion had occurred. In contrast, ferrets receiving a control lipopeptide were all infected by their cohoused infected cagemates. A single intranasal dose mediated protection over 24 h of close contact, making this lipopeptide an attractive prophylactic antiviral candidate (de Vries et al. 2021).

Next, these US researchers demonstrated that lipopeptide pretreatment protected transgenic mice expressing human ACE2 receptor from challenge with SARS‐CoV‐2 (80%–100% survival), prevented weight loss, and reduced viral RNA load in throat and lung. In practice, a prophylactic lipopeptide might be given repeatedly. Repeated exposure induced peptide‐specific antibodies in mice without reducing their antiviral effect. When mice with peptide antibodies were again treated with lipopeptides and challenged with a variant SARS‐CoV‐2, mice treated for a second time again survived a lethal viral challenge. When the lipopeptide was given therapeutically 8 h after the viral challenge, still half of mice survived SARS‐CoV‐2 challenge while all controls died. Interestingly, when lipopeptide‐treated mice surviving a first viral challenge were, after 3 weeks, again challenged with a second dose of SARS‐CoV‐2 without lipopeptide application, all mice survived the lethal challenge apparently because they had developed virus‐neutralizing antibodies from the first viral challenge. Neutralizing antibodies persisted for at least 6 months (Mougari et al. 2025).

### 1.3 Viral Maturation Inhibitors: M Protein

**FIGURE 3:**
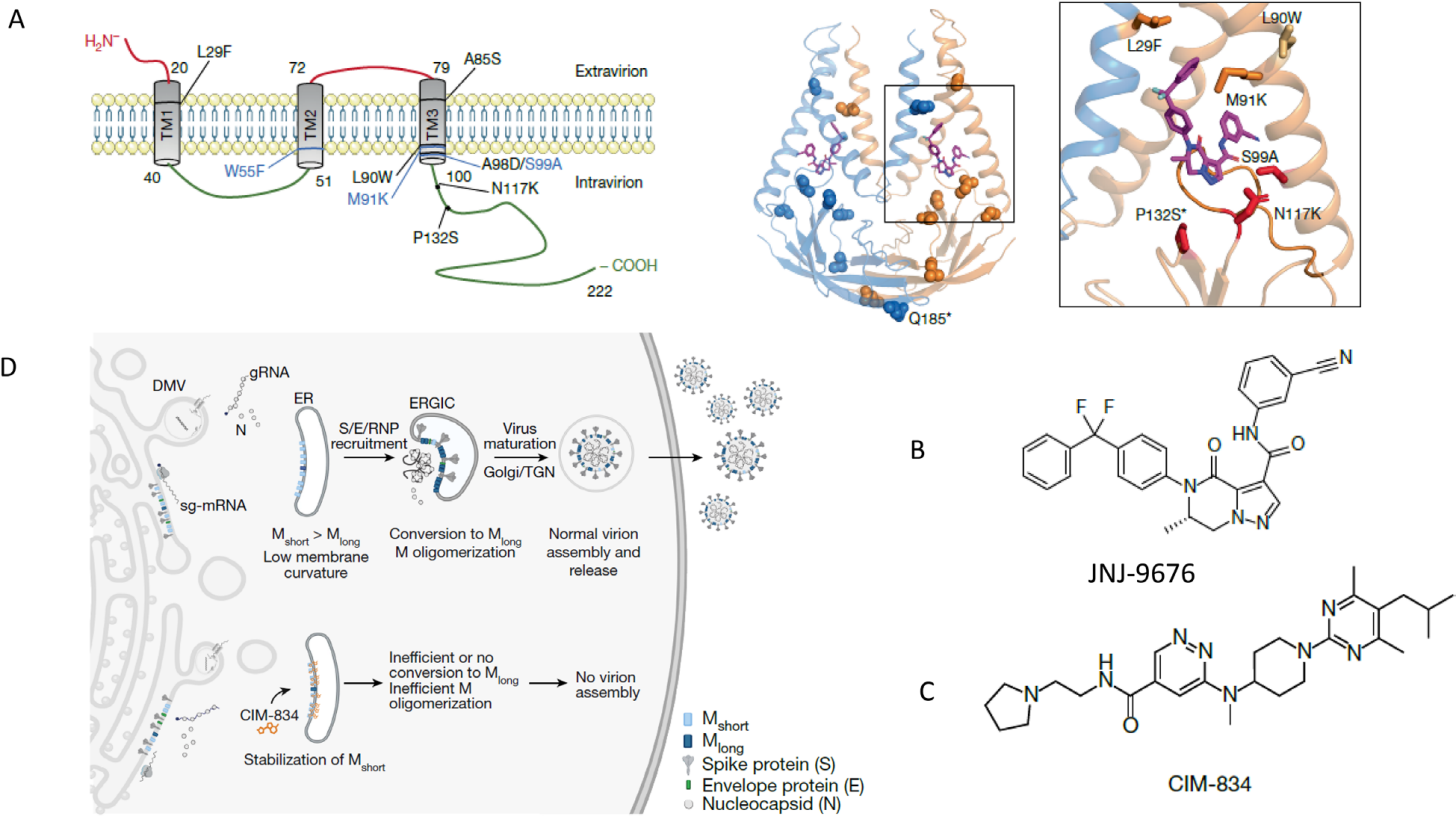
Viral maturation inhibitor binding to the viral M protein. (A) Left: The transmembrane structure of the SARS‐CoV‐2 M protein. Mutations conferring resistance to the inhibitor JNJ‐9676 are indicated in black. Center: 3D structure of the M protein with bound inhibitor JNJ‐9676 (violet stick model). Right: Amino acid contact sites for inhibitor JNJ‐9676 binding. (B) Structural formula of the M‐protein inhibitor JNJ‐9676. (C) Structural formula of the M‐protein inhibitor CIM‐834. (D) Proposed antiviral mechanism of CIM‐834 on maturation of intracellular progeny virus. DMV, Double‐membrane vesicles; ER, Endoplasmic reticulum; ERGIC, Endoplasmic reticulum‐Golgi intermediate compartment; gRNA, Genomic RNA; N, Viral nucleocapsid protein; S/E/RNP, Viral spike, envelope proteins and ribonucleoprotein; sg‐mRNA, Subgenomic viral mRNA; TGN, Trans‐Golgi network. Figure credit: (A, B) Van Damme et al. (2025) under a Creative Commons Attribution 4.0 International Licence. (C, D) Laporte et al. (2025) under a Creative Commons Attribution 4.0 International Licence.

Work from industrial and academic researchers revealed coronavirus M‐protein as an additional target for antiviral drug development. High throughput screening of a chemical library in a cell culture test at the pharmaceutical company Janssen identified compound JNJ‐9676 (Figure 3B) that showed inhibitory activity against different variants of SARS‐CoV‐2, bat and pangolin coronaviruses in the low nanomolar (nM) concentration range. Under single virus replication cycle conditions, time of addition experiments demonstrated that the compound acted on a late viral function. Drug‐resistant mutants were selected by serial passage experiments against increasing inhibitor concentrations and identified the viral M protein as target of JNJ‐9676 (Figure 3A). With reverse genetic engineering, three mutant sites conferred an about 100‐fold decrease in sensitivity towards JNJ‐9676. Coronavirus M protein has a short extra‐virion N terminal ectodomain, three transmembrane domains and a long intravirion C‐terminal domain (Figure 3A, left). The amino acid (aa) position of the three resistance mutations coincided with the binding site of the JNJ‐9676 inhibitor determined by cryo‐electron microscopy analysis of JNJ‐9676‐M protein complexes (Figure 3A, right). Notably, JNJ‐9676 binding induced a major conformational change in the M‐protein dimer. When given 1 h before viral challenge in a hamster SARS‐CoV‐2 infection model, JNJ‐9676 showed a dose‐dependent up to 4 log_10_ viral titre reduction in the lung as well as a reduced histopathological score. When given up to 48 h after viral challenge, JNJ‐9676 still showed a reduction in infection titers in the lungs (Van Damme et al. 2025).

An academic consortium again with Belgian lead scientists screened 350,000 small molecules for SARS‐CoV‐2 inhibitors in a cell culture test. It led to one compound with inhibitory activity in the μM concentration range. In a medicinal chemistry program, 500 analogues were tested leading to CIM‐834 inhibiting SARS‐CoV‐2 at 100 nM. CIM‐834 (Figure 3C) is a distinct chemotype than JNJ‐9676 but binds to a similar site of the M protein. In mice and hamsters, it had good oral bioavailability and lung distribution. In a therapeutic set‐up in an immunodeficient mouse SARS‐CoV‐2 infection model, CIM‐834 given 48 h after viral challenge reduced infectious virus titers by 3 log_10_, but showed no reduction in viral RNA (vRNA) concentrations. In a hamster SARS‐CoV‐2 infection model, CIM‐834 reduced infectious virus titers by 4 log_10_ in the lung and no virus was transmitted to uninfected cage littermates. A virus selected for growth in the presence of CIM‐834 showed a single aa change (P132S) in the M protein. Amino acid changes at positions 91 and 117 that conferred resistance to JNJ‐9676 had only a small impact on inhibition by CIM‐834. CIM‐834 inhibited the oligomerization of the M protein and the release of virus‐like particles. Thin‐section transmission electron microscopy revealed that CIM‐834 interfered with the virion assembly maturation step from the endoplasmic reticulum to the Golgi complex (Figure 3D). The transition is mediated by a structural change of the M protein from a short to a long conformation. This step increases the curvature of the membrane vesicle leading to virion assembly. CIM‐834 stabilizes the M short conformation and thus prevents the transition to the M long conformation needed for virion assembly (Laporte et al. 2025).

### 1.4 Viral mRNA Cap Inhibitors: NSP14 Inhibitors

**FIGURE 4:**
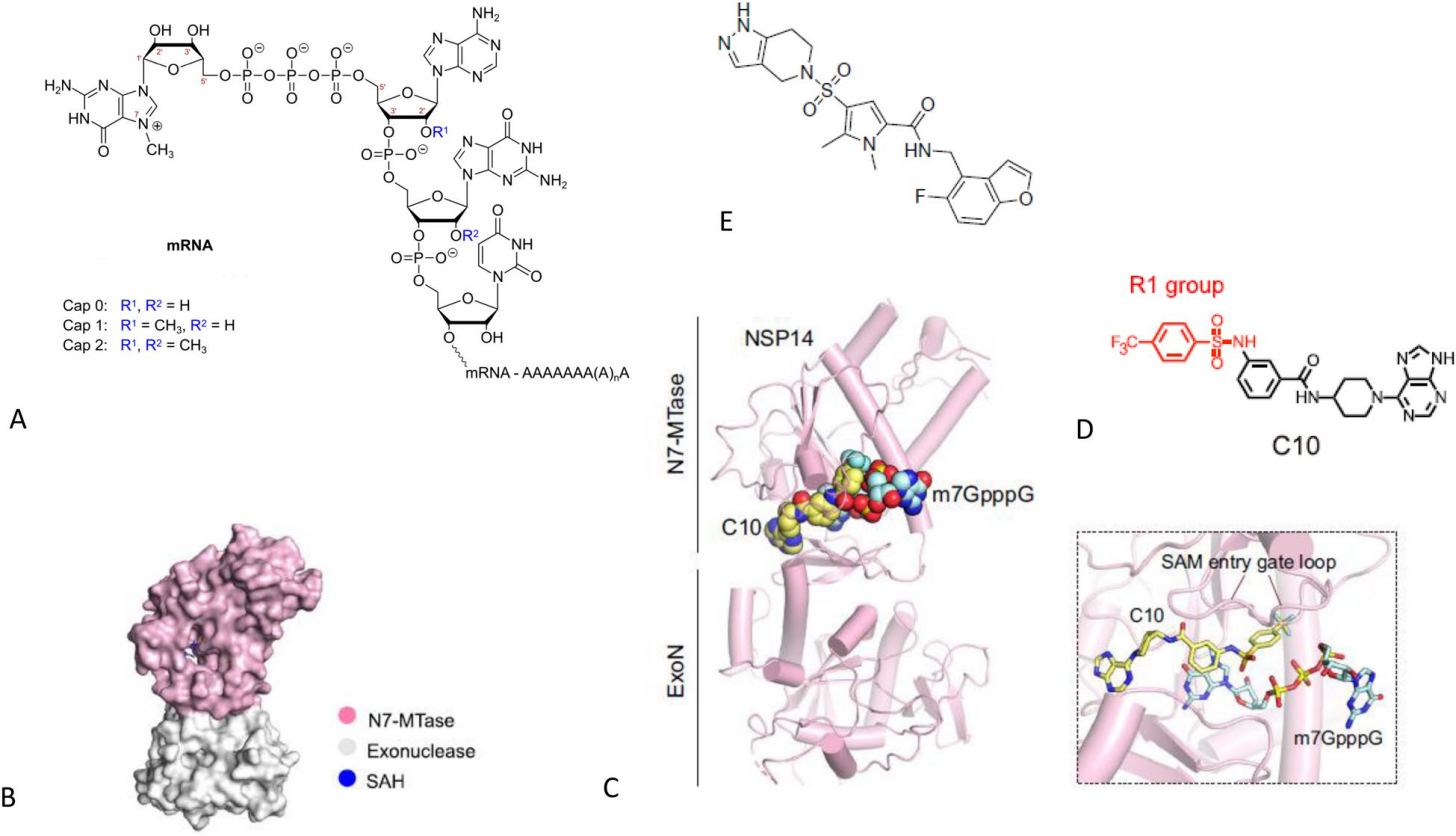
NSP14 and viral mRNA cap inhibitors. (A) mRNA cap 0, 1 and 2 structures with m^7^G cap poly A tail and nucleotide positions 1 and 2 with corresponding modifications. (B) 3‐D structure of NSP14 with N7 Methyltransferase and exonuclease domains. (C) NSP14 with bound C10 inhibitor next to m^7^GppG cap and the S‐adenosyl‐methionine entry gate loop. (D) Structural formula of the C10 inhibitor. (E) Structural formula of the TDI‐015051 inhibitor. Figure credit: Wikipedia Benff (A); Meyer et al. (2025) (E); Luo et al. (2025) (B–D) under a Creative Commons Attribution 4.0 International Licence.

Most viral and cellular mRNA molecules display the same cap structure, namely m7GpppN at the 5′ end. The cap fulfils several critical functions: it protects the mRNA from attack by 5′ exonucleases, increases translation efficiency and hides recognition sites for the intrinsic defence system of the cell. Complex viruses encode their own dedicated capping enzymes, providing additional targets for antiviral drugs. Some viruses, eg bluetongue orbivirus, have a unimolecular assembly line for capping, consisting of a multidomain enzyme removing the γ phosphate of the 5′ end of the viral mRNA (5′ triphosphatase), adding a guanosine in a 5′–5′ linkage (guanylyl transferase) and a methyl group from S‐adenosyl methionine (SAM) to the N7 position of the guanine base (guanine 7 methyltransferase, N7MT) forming cap 0. Subsequently, a 2′‐O‐methyl‐transferase (2′OMT) adds a methyl group to the first (cap 1) and second (cap 2) 2′ hydroxyl group of the ribose parts from nucleotides 1 and 2 of the mRNA (Figure 4A).

SARS‐CoV‐2 NSP14 consists of two distinct domains: a 3′–5′ exoribonuclease (ExoN) domain for viral RNA proofreading and a C‐terminal N7MT (Figure 4B). US researchers screened 430,000 chemical compounds for inhibitors of SARS‐CoV‐2 N7MT in a luminescence test. After subsequent counter screens, 28 compounds inhibited the enzymatic reaction at less than 10 μM. Medicinal chemistry hit‐to‐lead optimization yielded TDI‐015051 (Figure 4E) inhibiting at sub‐nanomolar concentration. Molecular docking indicated that the inhibitor occupied the guanine cap‐binding pocket of the MTase domain, forming a highly stable inhibitory ternary complex with N7MT and S‐adenosylhomocysteine (SAH), notably not the substrate S‐adenosylmethionine (SAM) but the end product for the methylation reaction. The inhibitor blocked SARS‐CoV‐2 infection at 60 nM concentration in cell culture. In mice, the drug achieved inhibitory concentrations maintained for 10 h after single oral application. In a mouse SARS‐CoV‐2 infection model, oral inhibitor application achieved a lung viral load reduction of 2 log_10_ comparable to the approved SARS‐CoV‐2 antiviral nirmatrelvir. The prophylactic dosing showed a 1 log higher viral load reduction than a treatment dosing starting at 12 h post‐infection (pi). The researchers addressed the mechanism of action for TDI‐015051 by selecting resistance mutations which were all located in the N7MT domain of the viral NSP14 protein. TDI‐015051 inhibited N7MT from both alpha‐ and beta‐coronaviruses, but did not interfere with the 2′‐OMT activity nor with the human cellular N7MT enzyme. TDI‐015051 induced 2000‐fold lower viral mRNA levels, a 35‐fold decreased mRNA translation, and an induction of interferon‐stimulated genes compared to control infections (Meyer et al. 2025).

Chinese researchers conducted a structure guided virtual screening of 150,000 compounds docked to the SAM binding site of NSP14 (Figure 4C). The top 20 fits were then tested in a luminescence‐based enzymatic assay. After optimization, the C10 lead (Figure 4D) showed a sub‐μM inhibitory concentration covering alpha‐ and beta‐coronavirus NSP14 but no interference with cellular enzymes. C10 demonstrated a SAM‐competitive inhibition mechanism. In cell culture tests, C10 inhibited SARS‐CoV‐2 and its variants. A resistance mutation was located to the catalytic pocket of the N7MT domain. Time of addition experiments identified for C10 post‐entry inhibition effects, affecting viral replication and viral translation. After intraperitoneal injection in mice, C10 maintained antiviral activity for 6 h. At a 120 mg/kg dose, C10 significantly reduced SARS‐CoV‐2 loads in the lungs of mice when given 1 h after viral challenge. However, dosing was delicate: at half this dose, viral load reduction was not any longer significantly reduced and at twice this dose, mice were killed by drug toxicity. The author concluded that further medicinal chemistry optimization of C10 is needed (Luo et al. 2025).

### 1.5 New Approaches Targeting RdRp (NSP12): RIBOTAC Inhibitors

Along the line of clinically approved RdRp inhibitors with 1′‐cyano group addition to the ribose unit such as remdesivir, obeldesivir and mindeudesivir (VV116, which contains further 2′ and 3′ derivation at the ribose unit), a new inhibitor was developed with 1‐cyanocytidine (CNC) and its 5‐isobutyril prodrug. CNC is rapidly absorbed after oral administration and has micro‐to‐nanomolar inhibitory activity against SARS‐CoV‐2 in cell culture. Following intraperitoneal injection, the prodrug induces up to 5 log_10_ reduction in viral RNA and total suppression of viral infectivity in the lung and at a 100 mg/kg dose it suppresses lung pathology. After oral administration, lesser, but still significant effects were observed (Amblard et al. 2025).

Another approach is the combination of approved COVID‐19 antivirals with the aim to reduce resistance development and to increase therapeutic efficacy. US researchers explored the latter effect by comparing monotherapy with either the RdRp inhibitor obeldesivir or Mpro inhibitor nirmatrelvir versus a combination of both antivirals in a mouse SARS‐CoV‐2 infection model. Combination therapy reduced the infectious viral load in the lung over that in monotherapy and achieved an amelioration of acute lung injury by histology which was not seen in monotherapy (Martinez et al. 2024).

Another interesting and promising approach targets RdRp with a chimeric inhibitor combining a targeting and an executing moiety. RNase L is an important mediator of innate immunity to viral infection. In its monomeric form, the ribonuclease domain is suppressed by intra‐molecular binding by ankyrin domains in RNase L. When the host cell detects double‐stranded RNA (dsRNA) as a warning sign of viral infection, interferon induces 2′,5′‐oligoadenylate synthetases which produce a series of short 2′,5′‐linked oligoadenylates (2–5A). These oligoadenylates bind the ankyrin domain, induce a conformational change in RNase L, which leads to RNase L dimerization and activation of its potent nuclease activity. However, 2–5A does not penetrate cell membranes and is rapidly degraded in serum. Through high throughput screening of chemical libraries, researchers identified small compounds that can enter cells, activate RNase L, are not cytotoxic and suppress the replication of diverse single stranded RNA viruses in cell culture, opening a door to novel broad‐spectrum antivirals (Thakur et al. 2007).

Chinese researchers argued that guided and controlled activation of RNase L could achieve more specific viral RNA degradation including that of SARS‐CoV‐2. Sequence selective viral antisense oligonucleotides (ASO) could both confer specificity and induce a basic level of cleavage for the target virus RNA by the cellular RNase H1. Combining ASO with an RNase L recruiter into an RNA chimera could increase the efficacy and specificity of such an antiviral. They combined a 15‐nucleotide long ASO targeting the SARS‐CoV‐2 envelope E or spike gene over a linker to a short polyA RNase L recruiter. This chimera achieved a more than 10‐fold titre reduction against pseudovirus in cell culture (Su et al. 2021).

The long and charged ASO moiety of the chimera represents pharmacological application problems. Therefore, simpler chemical alternatives were searched for the recognition of target sites. In addition, target RNA can be highly structured, making recognition of the target by ASO difficult, which is based on base‐pairing with a linear RNA. A US‐German research consortium screened a small compound library against a library of RNA 3D folds and came up with a collection of 10 classes of polycyclic aromatic compounds that displayed strong RNA binding capacities. However, binding of these polycyclic compounds to RNA represents mostly biologically silent interactions. Therefore, they linked the RNA‐binding molecule to a heterocycle that binds and locally activates RNase L to cleave the target RNA, thus generating a ribonuclease‐targeting chimera (RIBOTAC) (Tong et al. 2023).

**FIGURE 5:**
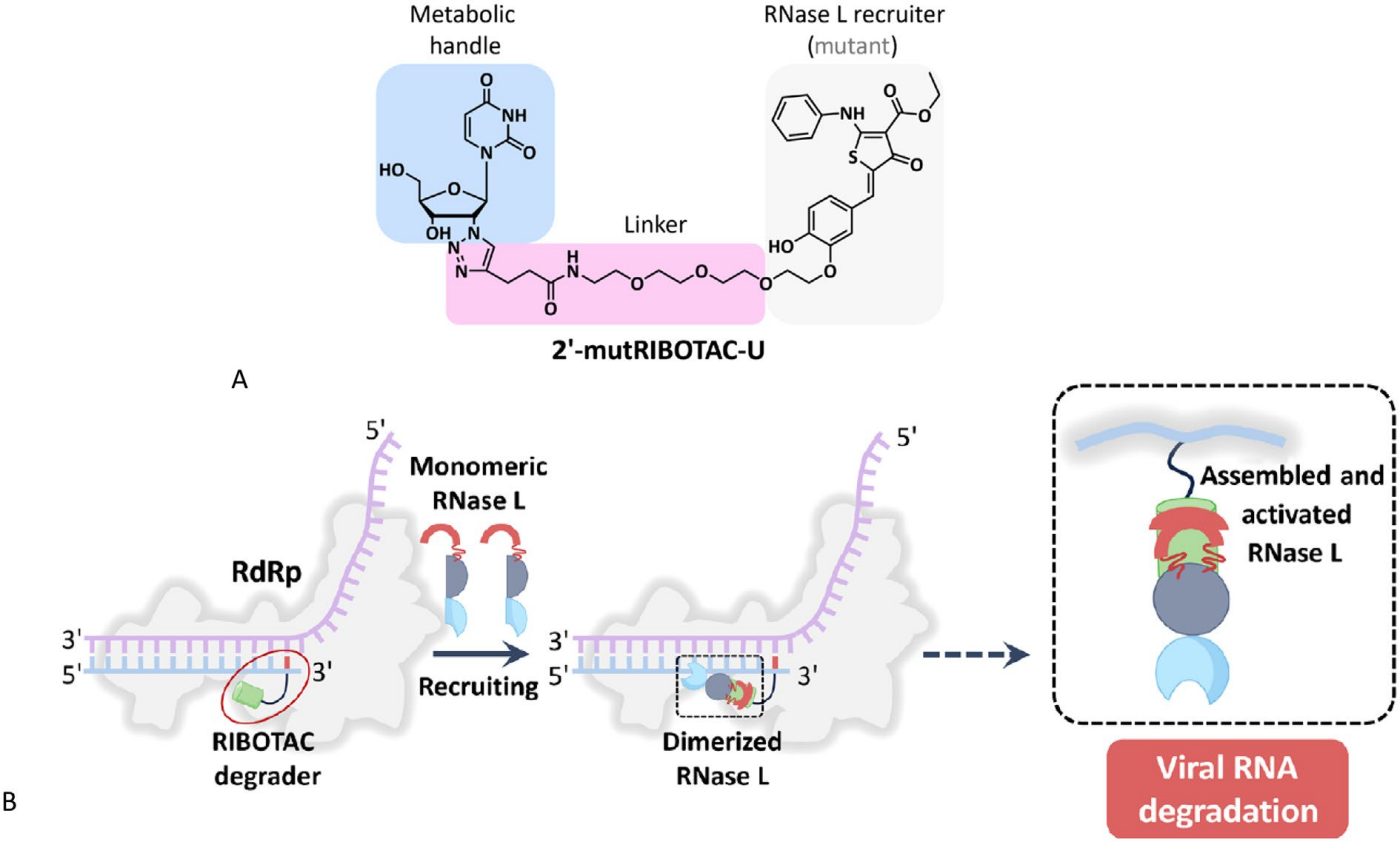
Ribonuclease‐targeting chimera (RIBOTAC) inhibitor of viral RdRp (NSP12). (A) Chemical structure of RIBOTAC inhibitor with metabolic handle binding as nucleotide analogue to SARS‐CoV‐2 RdRp, its linker and the RNase L recruiter. (B) Reaction scheme for RIBOTAC inhibitor: Left side‐ binding to the growing RNA chain at RdRp active site; center‐recruiting of RNaseL monomers and right‐ dimerization to active nuclease degrading the viral RNA. Figure credit: Min et al. (2024) under a Creative Commons Attribution Licence 4.0.

Chinese researchers explored an alternative approach for targeting SARS‐CoV‐2 with a RIBOTAC approach. They exploited the fact that SARS‐CoV‐2 RdRp, in contrast to cellular and mitochondrial polymerases, accepts nucleotide analogues that are the basis for approved COVID‐19 drugs such as remdesivir, molnupiravir, obeldesivir and VV16. Therefore, they synthesized a uridine derivative that contained a linker either attached to the 2′ ribose position or to the base part of uridine as a metabolic handle of the RIBOTAC, which binds to the active site of RdRp. The linker was attached to a small aromatic molecule consisting of three ring systems; one was a heterocycle serving as an RNase L recruiter (Figure 5A). The recruiter attracts the RNase L monomers, which dimerize and constitute the active nuclease which degrades the viral RNA (Figure 5B). The ribose‐modified RIBOTAC showed higher inhibitory activity in cell culture infections with SARS‐CoV‐2 than the base‐modified RIBOTAC constructs. The ribose‐attached RIBOTAC showed no adverse effects when injected intraperitoneally in hamsters. Following infection with SARS‐CoV‐2, the hamsters received daily 2′ ribose RIBOTAC injections, which decreased viral RNA in the lung and trachea, as revealed by RT‐PCR, reduced viral N protein expression as assessed by Western blotting and immunohistology. Vehicle‐treated hamsters manifested pronounced inflammatory responses and severe lung lesions, which were markedly less intensive in 2′ ribose RIBOTAC‐treated hamsters (Min et al. 2024).

Another target for RIBOTAC is the untranslated 5′ region (UTR) of SARS‐CoV‐2 RNA. Viral RNA in the virion is capped and polyadenylated and serves thus directly after entry as mRNA for translation. Also later in infection, the 5′ UTR leader serves as a binding site for the transcription complex which from there hops to the 5′ end of each individual gene for transcribing individual viral mRNAs. The 5′ UTR is thus a critical element that shows six stem‐loop secondary structures. US researchers had synthesized coumarin derivatives that bound to stem‐loop 5. When this coumarin derivative was linked to an RNase L recruiter and added to cell culture, it showed a > 95% inhibition of SARS‐CoV‐2 replication without toxic side effects (Tang et al. 2025).

### 1.6 NSP3: Papain‐Like Protease Inhibitors

**FIGURE 6:**
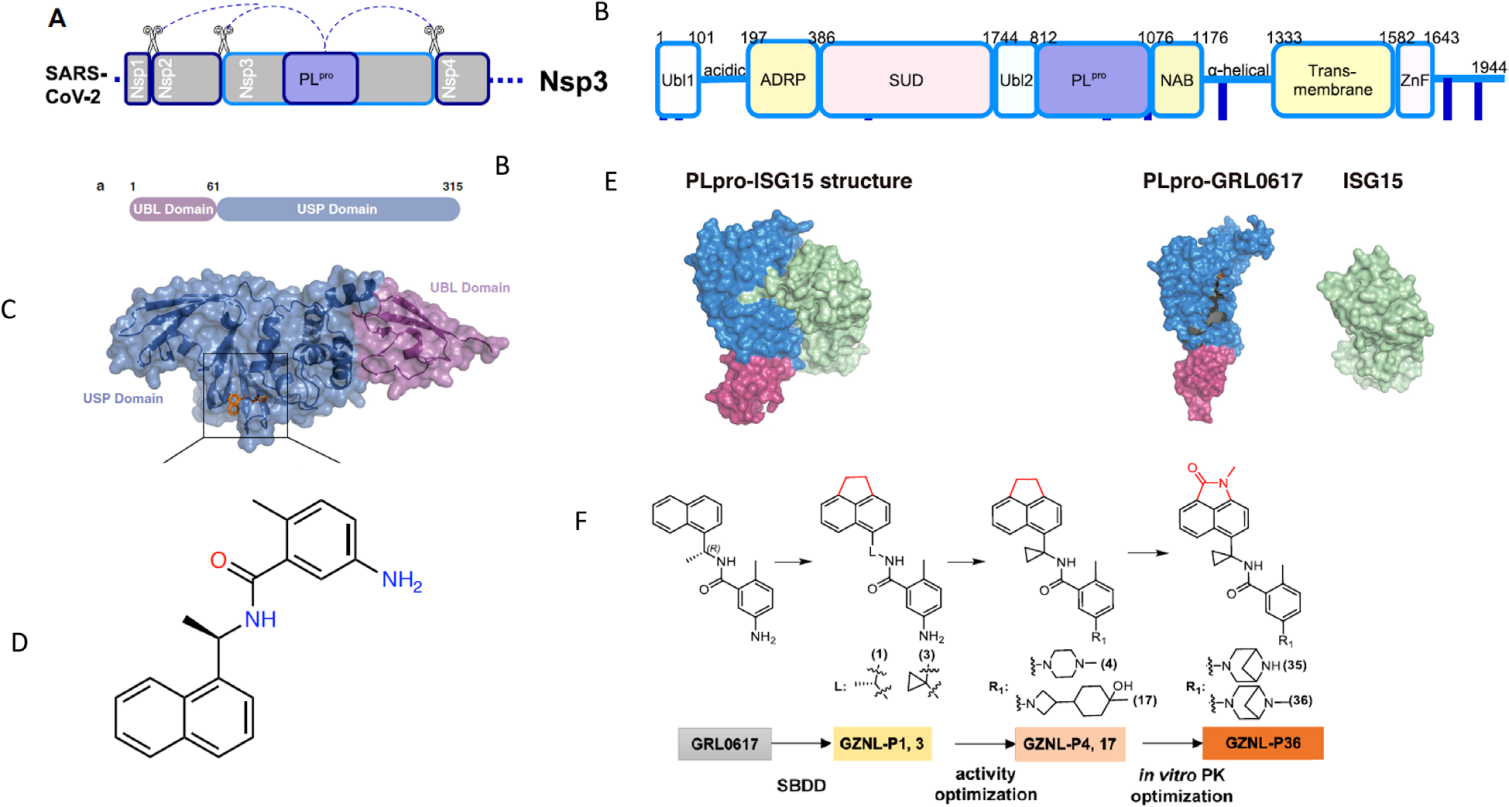
NSP3: Papain‐like protease inhibitors. (A) Location of the NSP3 gene at the 5′ end of the SARS‐CoV‐2 genome with indication of the position of the PL protease domain within NSP3 and the cleavage mediated by PL‐protease indicated by scissors. (B) Domain structure of NSP3 (Ubl1, 2: Ubiquitin‐like domain 1,2; PL^pro^: Papain‐like protease, deubiquitinase, deISGylase). (C) 3‐D Structure of part of the NSP3 protein with the N‐terminal ubiquitin‐like (UBL) domain and a C‐terminal ubiquitin specific protease (USP) domain with catalytic functions of cleaving ubiquitin or ISG15 modifications from host proteins involved in innate immunity. The position of the bound inhibitor GRL0617 is indicated in orange. (D) Structure of GRL0617 inhibitor. (E) Binding of the inhibitor to PL^pro^ USP prevents binding of ISG15. (F) Medicinal chemistry optimization of PLpro inhibitor starting with GRL0617. Figure credit: (A, B) Garnsey et al. (2024) under a Creative Commons Attribution Licence 4.0; (C–E) Fu et al. (2021) under a Creative Commons Attribution 4.0 International Licence; (F) Lu et al. (2024) Creative Commons Attribution‐Non Commercial‐No Derivatives 4.0 International Licence.

Two viral proteases play a vital role in the cleavage of the coronaviral ORF1a and 1b polyproteins, liberating the sixteen non‐structural proteins NSP1 to NSP16 of SARS‐CoV‐2. NSP3 is a large and complex protein containing several domains with distinct functions (Figure 6B), including a papain‐like protease (PLpro) domain that liberates NSP1 to 3 by proteolytic cleavage (Figure 6A). NSP5 is a much smaller chymotrypsin‐like protease (CLpro); it represents the major SARS‐CoV‐2 protease (Mpro), which releases NSP 4 to 16 and is the target of the approved COVID‐19 antiviral nirmatrelvir.

The multidomain NSP3 protein also suppresses the host immune response through cleaving ubiquitin and interferon‐stimulated gene 15 (ISG15) and thus interfering with the innate immune response to a viral infection. NSP3 also binds ssRNA and the viral N protein and it removes ADP‐ribose additions from proteins. This functional complexity explains why PLpro lagged behind Mpro drug development. At the same time, its multifunctionality makes it an attractive target for antivirals.

In the early phase of the COVID‐19 pandemic, Chinese researchers screened a library of 2040 drugs approved by health authorities for PLpro inhibitory activity. Only modest antiviral activity was associated with seven selected compounds. Therefore, the researchers focused on GRL0617 as lead compound (Figure 6D), an inhibitor of SARS‐CoV PLpro which shares 83% aa sequence identity with SARS‐CoV‐2 PLpro. GRL0617 inhibited the deISGylation (ISG15 cleavage) activity of PLpro as target effect. Crystallographic studies revealed that GRL0617 is a non‐covalent inhibitor of the ubiquitin specific proteases (USP) domain of PLpro (Figure 6C). NMR data indicate that GRL0617 blocks the binding of ISG15 C‐terminus to PLpro (Figure 6E). GRL0617 thus interferes with the inhibition of the cellular innate antiviral response mediated by PLpro (Fu et al. 2021).

Chinese scientists modified GRL0617 by structure‐based drug design leading to PLpro inhibitor GZNL‐P36 (Figure 6F). In cell culture infections, it showed comparable antiviral activity against SARS‐CoV‐2 and its variants and common cold‐associated human coronaviruses as the approved CLpro inhibitor ensitrelvir. The inhibitor decreased viral lung titers in SARS‐CoV‐2 infected mice in a dose‐dependent way from 100‐fold to > 10,000‐fold. At a comparable dose, reduction was however 10‐fold less than that observed with ensitrelvir, a CLpro inhibitor. Lung histopathology was decreased, as well as the transcription of pro‐inflammatory cytokines and chemokines (Lu et al. 2024).

**FIGURE 7:**
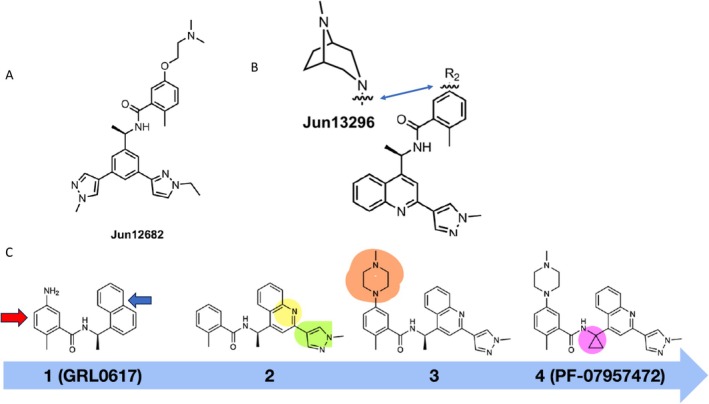
Further Papain‐like protease inhibitors. (A) Jun12682 developed by Tan et al. (2024). (B) Jun13296 developed by Jadhav et al. (2025). (C) Inhibitor amelioration by medicinal chemistry in Garnsey et al. (2024). Naphthalin (blue arrow) was replaced by quinoline (yellow) and an *N*‐methyl pyrazole derivative (green). The aniline moiety (red arrow) got a methyl piperazine substituent (brown) and the linker a cyclopropyl group (violet). Figure credit: (A, B) Jadhav et al., licensed under a Creative Commons Attribution‐Non Commercial‐No Derivatives 4.0 International Licence; (C) Garnsey et al. (2024) under a Creative Commons Attribution Licence 4.0.

US researchers synthesized compounds that bound to an ubiquitin binding site of NSP3. Structural analysis of the complex and optimization of the inhibitor structure led to a compound that displayed in vitro antiviral activity in the sub‐μM concentration range. The lead inhibitor Jun12682 (Figure 7A) blocked the antagonizing action of PLpro on the innate immune reaction as mechanism of action. The researchers demonstrated good oral bioavailability for this inhibitor, a sufficiently long half‐life to maintain antiviral plasma activity in mice for 7 h and stability in human microsomes (suggesting that it could be applied without ritonavir booster needed for nirmatrelvir, a CLpro inhibitor). In a lethal SARS‐CoV‐2 mouse infection model, the inhibitor attenuated weight loss in infected mice compared to vehicle control and increased survival to 70% and 100% for 5 and 3 days of treatment, respectively, while all vehicle‐treated mice died. Viral lung titers decreased 10‐fold and cellular cytokines and chemokines were substantially reduced. Jun12682 was active against SARS‐CoV‐2 variants and a nirmatrelvir‐resistant viral mutant (Tan et al. 2024).

In structural analysis of Jun12682‐PLpro complexes, the inhibitor binds to the same site as GRL0617 in the BL2 loop of PLpro and extends to a critical Val70 site in the ubiquitin binding region. Stepwise lead optimization, which included replacement of the naphthalene by a quinoline ring system, led to Jun13296 (Figure 7B), which maintained virus inhibitory plasma concentrations after oral administration for 8 h in mice and showed no off‐target activity against host proteases. When given 1 h pi to SARS‐CoV‐2 infected mice, Jun13296 reversed initial weight loss and achieved 90% survival in lethal infections. Even at a lower dose Jun12682 assured 40% survival. Viral titers, viral antigen and inflammatory cytokine expression were reduced 10‐fold by Jun13296 (Jadhav et al. 2025).

Pfizer scientists reported on a machine learning–driven scan of large regions of chemical space starting with a compound that showed weak antiviral activity against SARS‐CoV‐2 in cell culture infections. The parent compound combined an aniline with a naphthalene ring. By introducing a substituent to the aniline moiety and replacing the naphthalene ring and adding a substituent to the linker led to a compound that showed inhibitory activity in the sub‐μM range (Figure 7C). The active compound bound the substrate but not the active site of the PLpro enzyme. Antiviral activity was correlated with inhibitory activity against the proteolytic activity of PLpro. Pharmacological tests revealed no off‐target activity and good bioavailability in a panel of animals including monkeys. Mice treated with this compound 4 h after SARS‐CoV‐2 challenge experienced no weight loss and showed, at a 150 mg/kg dose, a superior reduction of viral lung titers than 1000 mg/kg nirmatrelvir and prevented lung pathology. The industrial scientists underlined the utility of machine learning–enabled medicinal chemistry which compressed the time needed for antiviral development which is crucial in face of a rapidly developing viral pandemic such as COVID‐19 (Garnsey et al. 2024).

Australian researchers screened a library of 400,000 small chemical compounds for inhibitory activity of ubiquitin cleavage by PLpro which identified 16 hits. Medicinal chemistry improved the cell culture viral inhibitory activity into the sub‐nM range. The compounds demonstrated binding to a hydrophobic pocket not occupied by all previous PLpro inhibitors. The WEHI‐P8 candidate showed antiviral activity in mice after oral application without ritonavir boost. WEHI‐P8 reduced dose‐dependently viral lung titers to a greater extent than nirmatrelvir/ritonavir treatment, prevented lung pathology and reduced weight loss and proinflammatory cytokines. It was as effective in treatment as in prophylactic application. The Australian scientists developed a SARS‐CoV‐2 mouse infection model that mirrored some aspects of PASC (long‐Covid) for which treatment options are currently lacking. In their model, mice cleared the infection within a week, but still showed histological signs of disease 1–3 months after acute infection. WEHI‐P8 treated animals showed reduced post‐acute signs of haemorrhage and immune cell infiltrates in the lungs as well as reduced neuroinflammation. Treated mice showed better performance in behavioural, memory and cognitive tests than nirmatrelvir/ritonavir treated mice (Bader et al. 2025).

## 2 Targeting Host Functions Used by Viruses

There are two fundamental groups of antivirals: direct‐acting antivirals (DAAs) that target viral proteins or viral gene functions and host‐directed antivirals (HDAs) that inhibit host functions needed for the viral infection process. Since RNA viruses have small genomes—coronaviruses with 30‐kb genomes are the largest RNA viruses—many functions needed for viral multiplication are provided by the much larger host genome. Host proteins therefore also provide a much greater target size for antivirals. This not only opens greater opportunities for drug companies but offers another advantage. A constant problem for antiviral development—as for antimicrobial drugs in general—is resistance development by microbial pathogens to the drug since the pathogen experiences strong selection forces and particularly RNA viruses display high mutation rates. If host genes are targeted by antivirals, drug resistance is unlikely to occur. On the flip side of HDAs are of course much higher risks of toxicity if important host functions are blocked. Safety of such drugs is therefore a major concern. This concern is somewhat alleviated by the fact that many important cellular functions are redundantly fulfilled and viruses will hijack only one protein. In addition, HDAs will only be applied transiently during the short period of a treatment in the acute infection phase. What about the prospect for HDAs against coronaviruses?

### 2.1 Host Protease Inhibitors

Host proteases that mediate the proteolytic activation of the SARS‐CoV‐2 spike protein, which is necessary for the entry process of viruses into the cell, are a suitable target for HDAs. The spike protein S experiences two sequential proteolytic cleavages by host proteases. The first spike cleavage occurs at the S1/S2 site, mediated by host furin‐like proteases. The second cleavage occurs at the S2′ site adjacent to the fusion peptide, which triggers the fusion event. This cleavage is mediated by the host serine protease TMPRSS2 (trans‐membrane protease serine 2). Exploring inhibitors of TMPRSS2, which cleaves after Arg or Lys, offers another advantage: these viral cleavage sites are well conserved among SARS‐CoV‐2 variants.

**FIGURE 8:**
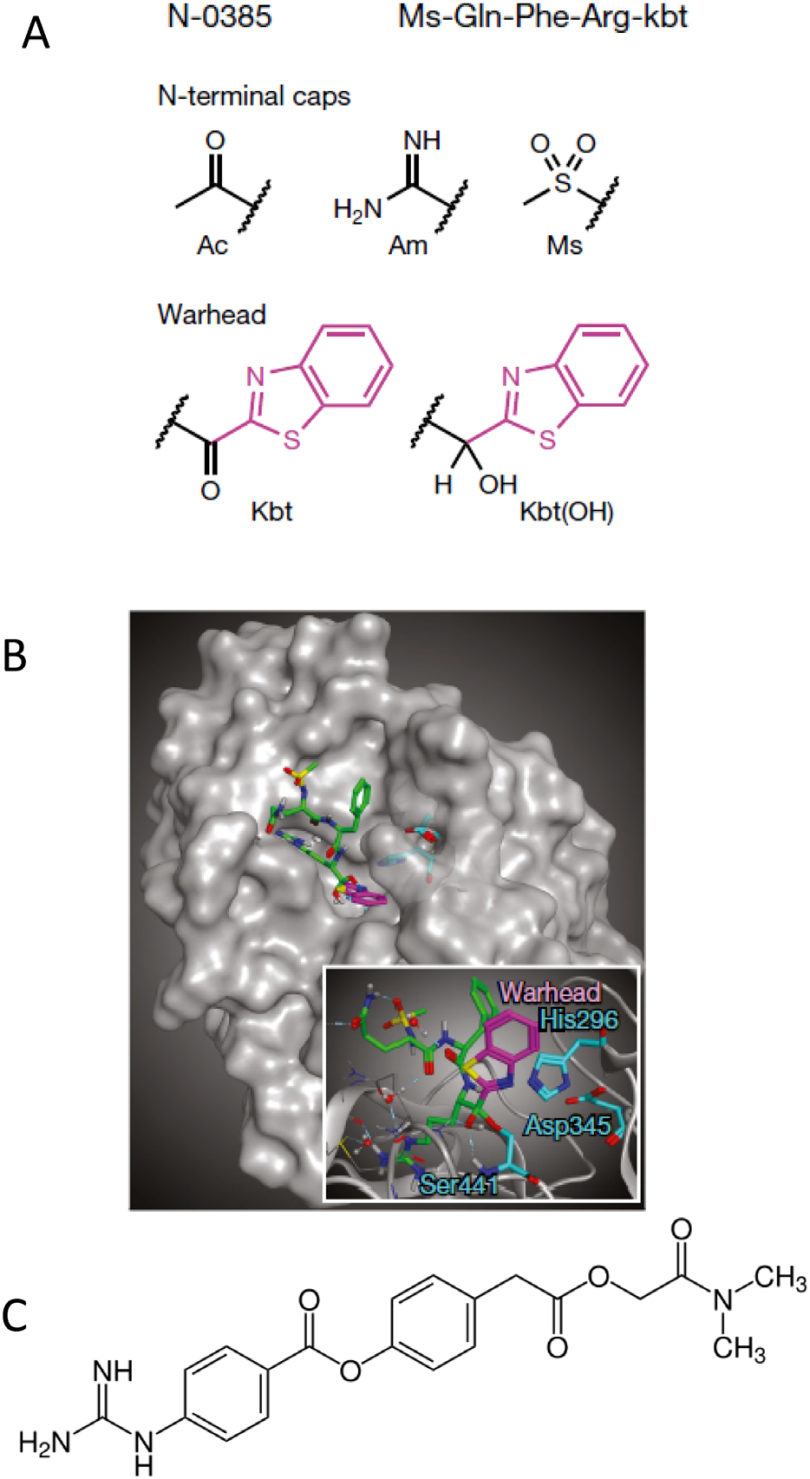
Ketobenzothiazole‐based peptidomimetics as host protease TMPRSS2 inhibitors. (A) The peptide sequence, N‐terminal caps and the ketobenzothiazole warhead of the N‐0385 inhibitor. (B) Docking of N‐0385 (green; warhead in purple) in the binding pocket of TMPRSS2. Residues of the catalytic triad are shown in cyan. (C) Structure of the approved pancreatitis drug camostat. Figure credit: (A, B) Shapira et al. (2022) under a Creative Commons Attribution Licence 4.0. C: Wikipedia.

Based on their experience with HDA development against influenza viruses, Canadian researchers explored peptidomimetic tetrapeptides as TMPRSS2 inhibitors by an in vitro activity test. The most active compound was N‐0385 (Figure 8A), a Gln‐Phe‐Arg tripeptide capped at its N‐terminus by a mesyl group and displaying at its C‐terminus a pharmacological warhead with an active keto group fitting into the active site of TMPRSS2 (Figure 8B). In cell culture infections, N‐0385 exhibited SARS‐CoV‐2 inhibitory activity in the single digit nM concentration range and no cytotoxicity. In colon organoids treated with N‐0385, production of SARS‐CoV‐2 was not observed. When giving N‐0385 by intranasal application for 8 days in a mouse model of SARS‐CoV‐2 infection, starting 1 day before viral challenge, 70% of the treated mice survived the infection compared to none in controls. Surviving mice showed weight gain and no pathological signs and no viral infectivity was detected in the lungs. N‐0385 also protected mice against the Delta variant of SARS‐CoV‐2. Even a single intranasal dose given 12 h after viral challenge reduced the viral infectivity in the lung and attenuated the weight loss (Shapira et al. 2022).

In cell culture infection, N‐0385 is 5‐fold more active as an antiviral than camostat mesylate (Figure 8C), an approved drug against pancreatitis, which inhibited TMPRSS2 as a mechanism of action. N‐0385 prevented pseudotyped MERS, SARS‐CoV and SARS‐CoV‐2 entry in cell culture and also inhibited authentic SARS‐CoV‐2 infection (Hoffmann et al. 2020). However, in a clinical trial with 205 COVID‐19 patients treated on a 2:1 basis with camostat mesylate or placebo within 48 h of hospitalization, no effect of camostat mesylate on median time to clinical improvement, 30 day mortality, or median change in viral load from baseline were detected in comparison with placebo (Gunst et al. 2021). A meta‐analysis of 9 RCT involving 1623 COVID‐19 patients demonstrated that camostat mesylate did not accelerate clinical recovery (measured as clinical resolution of symptoms and time to symptom improvement) or the viral clearance (time for PCR negative tests) (Khan et al. 2024). It is thus unlikely that N‐0385 can be used as antiviral against coronaviruses.

### 2.2 Sphingomyelin Biosynthesis Inhibitor

Several RNA viruses such as coronaviruses, flaviviruses and bunyaviruses use lipid droplets derived from the endoplasmic reticulum as sites for virus replication. Substances interfering with lipids of this compartment are thus potential antiviral drugs against these RNA viruses. Chinese researchers conducted a genome‐wide CRISPR‐Cas9 screen in human cells which were then infected with an emerging tickborne bunyavirus from China. Uninfected cells were purified by FACS and single guide RNA was sequenced, identifying SGMS1, encoding a key enzyme of the sphingomyelin biosynthesis pathway. Knock‐out of the gene reduced viral replication while trans‐complementation with the gene restored viral replication. Early viral infection steps were unaffected in knock‐out cells but vRNA replication was reduced. Also, the replication of SARS‐CoV‐2, dengue virus (a flavivirus), and an arenavirus were reduced in these knock‐out cells, pointing to the dependence of a wide range of RNA viruses on this lipid compartment for vRNA synthesis. In fact, the RNA‐dependent RNA polymerases (RdRp) from these viruses bound a specific sphingomyelin. SGMS1^−/−^ mice showed reduced fatality, reduced viremia, and less spread to organs when infected with the bunyavirus. The researchers selected a small chemical compound WYFA15 fitting to a binding pocket near the catalytic site of RdRp that inhibited viral replication at 7 μM concentration and displayed cytotoxicity at 180 μM. In a lethal mouse infection model with bunyavirus, WYFA15 decreased mortality and viremia. Mice treated with WYFA15 and infected with SARS‐CoV‐2 also showed decreased viral lung titers and mitigated lung pathology (Han et al. 2024).

### 2.3 Elongation Factor Inhibitor

A large consortium of US and French researchers cloned and expressed in a human cell line 26 of the 29 SARS‐CoV‐2 proteins to which a Strep tag was added. These proteins were used as baits for association with human proteins. The protein complexes were affinity purified by a Strep antibody and characterized by mass spectrometry. Overall, 332 human proteins associated with the 26 SARS‐CoV‐2 proteins. Gene ontology characterization revealed an enrichment of host proteins involved with the endomembrane compartment or vesicle trafficking. Fittingly, the highest expression levels of the caught cellular proteins were detected in the lungs and their expression increased in virus‐infected cells. The SARS‐CoV‐2 protein interactome showed highest overlap with that of the West Nile virus, another RNA virus of the Flavivirus family. SARS‐CoV‐2 proteins also interacted prominently with proteins of the innate immune pathways and the host translation machinery. For the 332 interacting host proteins, they identified 66 druggable host proteins targeted by 69 investigational drugs or preclinical molecules. Two cell culture viral inhibition tests were conducted based on either immunofluorescence assays of viral proteins or RT‐PCR viral RNA detection. Strong antiviral effects were seen for zotatifin, interfering with the viral NSP9‐host elongation factor eIF4A interaction and ternatin‐4, interfering with the viral NSP13 and elongation factor eEF1A interaction. Notably, the eEF1A inhibitor aplidin/plitidepsin was used clinically in patients with multiple myeloma, making it an attractive drug for further antiviral testing against coronaviruses. Drugs inhibiting host proteins that interact with viral proteins hold promises for panviral therapies. Using repurposed FDA approved drugs allows in addition rapid initiation of clinical trials under emergency situations of viral outbreaks (Gordon et al. 2020).

Subsequently, US researchers investigated the antiviral activity of plitidepsin in preclinical tests. Plitidepsin is a cyclic depsipeptide originally isolated from a Mediterranean marine tunicate (*Aplidium albicans*). In primary human lung cells, plitidepsin showed SARS‐CoV‐2 inhibitory activity in the low nM concentration range; cytotoxicity was observed at 20‐fold higher concentrations. Time‐of‐addition assays demonstrated antiviral activity when added 4 h after infection. Decreased antiviral activity was observed in cells expressing an elongation factor eEF1A with a single amino acid replacement, which identified eEF1A as a target for plitidepsin action. Silencing of eEF1A protein expression with small interfering RNA (siRNA) reduced the viral negative strand RNA synthesis and viral N protein expression. When giving plitidepsin in three daily doses starting 2 h before challenging mice with SARS‐CoV‐2, it reduced the viral titers by 100‐fold compared to controls and decreased lung inflammation. In these mouse experiments, plitidepsin achieved the same inhibitory effects as a 50‐fold higher concentration of the FDA‐approved COVID‐19 drug remdesivir, an RdRp inhibitor (White et al. 2021).

Detailed molecular analysis revealed that plitidepsin inhibited already the early translation of the positive strand viral genome thus interfering with the build‐up of the double membrane vesicle formation needed for later viral transcription and replication. Plitidepsin's lack of toxicity to the host cell was explained by a preferential inhibition of viral over host protein translation. This specificity was explained by an upregulation of eIF4G2 by plitidepsin, which is required to initiate cap‐independent host mRNA translation. Plitidepsin showed panviral inhibitory activity against Coronaviridae, Flaviviridae, Pneumoviridae and Herpesviridae. In contrast, viruses that initiate translation differently, for example, by IRES (Internal Ribosome Entry Site) reading such as Retroviridae were not affected (Molina Molina et al. 2025).

Other researchers also reported plitidepsin inhibitory effects at low nM concentrations against a human respiratory syncytial virus, a negative strand Pneumovirus. However, depending on the cell line, plitidepsin affected the translation of both viral and host proteins in a similar manner, calling for caution in its clinical use (Estampes et al. 2025).

In preparation of a randomized open phase I clinical trial, an international consortium documented antiviral activity of plitidepsin against a wide range of SARS‐CoV‐2 variants. Then 46 adults hospitalized with COVID‐19 were treated with increasing daily doses of 1.5–2.5 mg plitidepsin. Once daily doses maintained viral inhibitory plasma concentration of the drug. More than 100‐fold viral load decreases were observed from the 4th day of treatment. Two patients showed grade 3 adverse events (hypersensitivity and diarrhoea) (Varona et al. 2022).

A phase III RCT was conducted in Spain with 205 hospitalized COVID‐19 patients needing oxygen support. They were randomized on two different doses of plitidepsin (1.5 and 2.5 mg daily) or standard therapy. Initially, 600 patients were planned for enrolment but decreasing patient numbers led to a premature stop of the trial. Median time to sustained withdrawal of oxygen supplementation, the primary trial outcome, was 5 days in plitidepsin‐treated vs. 7 days in control patients, but the difference was statistically not significant (*p* = 0.063). The lack of significance might be due to an underpowered trial caused by an early stop. Plitidepsin was generally well tolerated (Landete et al. 2024).

### 2.4 Outlook

One might argue that dedicating substantial research efforts to new coronavirus antivirals is of questionable use when COVID‐19 has lost most of its public health threat and societal burden. Such reasoning is however shortsighted. COVID‐19 might return with a virulent variant as has historically been observed in the Russian flu pandemic of 1889 which might have been an earlier coronavirus pandemic (Brüssow and Brüssow 2021) and which was followed 10 years later by a resurge (Brüssow 2021). Even if the Russian flu pandemic was caused by an influenza virus, an even more deadly now definitive influenza virus pandemic followed with the Spanish flu from 1918. Sadly, after a viral pandemic might just be before the next viral pandemic. One is therefore well advised to support antiviral research. This should be a health priority because there are only few approved antivirals when compared to antibiotics. Even the more numerous antibiotics confront now a resistance crisis. Resistance development seems to be inevitable when treating microbial infections with drugs targeting bacteria or viruses. To confront resistance problems, it is important to extend the range of protein targets for antivirals. As shown in this review, new druggable proteins of coronaviruses have been described. Structural proteins such as the coronavirus spike protein were targeted with N‐glycan binders and lipoproteins to interfere with receptor binding and cell fusion, respectively. Drugs interacting with the viral structural M protein interfere with viral maturation steps. Inhibitors against NSP14 involved in viral mRNA cap synthesis were explored as well as inhibitors of NSP3, a protease mediating the subversion of innate immune reaction against the virus. Even for NSP12 (RdRp), a pharmaceutical exploited target for approved antiviral drugs, innovative alternative biotechnological approaches for antivirals have been introduced with ribonuclease‐targeting chimeras (RIBOTACs). A fascinating aspect of antiviral research against coronaviruses is the prospect that some approaches show broad inhibitory activity against several groups of coronaviruses and some even indicate efficacy against diverse RNA viruses. Exploiting these cross‐reactive activities of antivirals will be one of the most promising approaches for pandemic preparedness efforts. Having at least a conceptional basis for cross‐reacting antivirals will accelerate the development of antivirals against the unknown viral agent of the next pandemic.

Another important aspect of the reviewed coronavirus antiviral research is the extension to small molecules interfering with host proteins that viruses need to complete their cellular multiplication cycle. Currently, there are only a handful of approved antivirals targeting host functions. Research in this field is urgently needed because host protein‐targeting antiviral drugs are unlikely to suffer from resistance development. Resistance development is a major reason that discouraged investments of the pharmaceutical industry into antibiotic development. Also, for the academic researcher, this field will be a fruitful area. Not only will it provide probes for a deeper understanding of cell biology, for virologists it opens a wide new field of research. Virologists have differentiated virions from virus; the former are the extracellular viral particles with their genomes and structural components, while the latter are a much more complex entity describing the network of viral gene/protein interactions with cellular genes/proteins needed for viral multiplication in the cell, which are not directly encoded in the viral genome. The interactome work as conducted by Gordon et al. (2020) suggests that coronaviruses extend their genetic capacities at least 10‐fold by recruiting cellular proteins for their replication. The cellular functions collaborating in viral multiplication can be seen as an extended genotype of viruses (DeLong et al. 2022). Virology, seen from the perspective of the virion, is thus just the tip of the iceberg. Below the surface, there is a lot of interaction with the host cell genome and proteome. Virus research will therefore soon become a cell biology of the infected cell, as already suggested by the virocell concept (Forterre 2013). Screening small chemicals as antiviral inhibitors of cellular functions will thus not only provide lead compounds for industrial drug development but also important research tools to define the virocell concept in molecular detail.

## Author Contributions

**Harald Brüssow:** conceptualization, writing – original draft, investigation, writing – review and editing.

## Funding

The author has nothing to report.

## Conflicts of Interest

The author declares no conflicts of interest.

## Data Availability

The author has nothing to report.
